# Evidence Based Alternative Medicines in Pain Management

**DOI:** 10.1155/2015/313821

**Published:** 2015-08-05

**Authors:** Haroon Khan, Bruno Eto, Vincenzo De Feo, Anwar-Ul-Hassan Gilani

**Affiliations:** ^1^Department of Pharmacy, Abdul Wali Khan University, Mardan 23200, Pakistan; ^2^Transcell-lab P.245 A, Faculté de Médecine Xavier Bichat, 16 rue Henri Huchard, 75018 Paris, France; ^3^Department of Pharmacy, University of Salerno, Fisciano, 84084 Salerno, Italy; ^4^The Aga Khan University Medical College, Karachi 3500, Pakistan

Pain is undoubtedly unpleasant sensation that affects the life style of a large population around the world. Several therapeutic approaches are used for the effective management of pain including use of drugs and alternate measures. This special issue describes the various events in traditional/alternative systems treatment for the management of painful condition.

The article entitled “Laser Acupuncture for Postoperative Pain Management in Cats” demonstrated that laser acupuncture significantly reduces postoperative analgesic requirements in cats undergoing ovariohysterectomy. Low back pain (LBP) is one of the most common complaints in the emergency department (ED) but the available therapies are having serious side effects. The clinical study in an article “Efficacy and Safety of Acupuncture for Acute Low Back Pain in Emergency Department: A Pilot Cohort Study” showed that acupuncture offered significant pain reduction in low back pain in patients treated in emergency department without any side effect.

Postoperative pain is very frequent and hard to treat. Dezocine is an opioid *µ* receptor partial agonist/antagonist widely used because of its potency and safety. The clinical studies in the article “Dezocine Prevents Postoperative Hyperalgesia in Patients Undergoing Open Abdominal Surgery” showed that dezocine offers a significant antihyperalgesic and analgesic effect in patients undergoing elective open gastrectomy for up to 48 h postoperatively.

The review article “Complementary and Alternative Medicine for the Management of Cervical Radiculopathy: An Overview of Systematic Reviews” concluded that current systematic reviews showed potential advantages to CAM for CR in alleviating neck pain or related symptoms. Due to the frequently poor methodological quality of primary studies, the authors suggested that the conclusions should be treated with caution for future clinical practice.

Approximately 75–90% of cancer patients experienced pain while 50% of patients are not satisfied with pain treatment that could affect the cortisol level. The article of this issue “Decreased Cortisol and Pain in Breast Cancer: Biofield Therapy Potential” exhibited marked reduction in cortisol concentration treated with bioenergy, suggesting reduction in stress due to pain.

The review article of Lo et al. “The Effects of Acupuncture on Cerebral and Muscular Microcirculation: A Systematic Review of Near-Infrared Spectroscopy Studies” based on available literature believed that research utilizing Near-Infrared Spectroscopy Studies to investigate the hemodynamics of acupuncture presently lacks in scope and quality. Improved designs, for example, placebo-controlled, randomized trials, and standardized intervention reporting could enhance study quality.

In this research study entitled “Involvement of Cholinergic and Opioid System in *γ*-Terpinene-Mediated Antinociception” the gamma terpinene (*γ*-TPN), a monoterpene, when studied for antinociceptive effect showed significant antinociceptive activity in various pain assessment models. The results suggest that the *γ*-TPN (p.o.) produced antinociceptive effect in models of chemical nociception through the cholinergic and opioid systems involvement.

A research article “Transcutaneous Electrical Acupoint Stimulation Improves the Postoperative Quality of Recovery and Analgesia after Gynecological Laparoscopic Surgery: A Randomized Controlled Trial” based on prospective, randomized, double-blind, placebo-controlled clinical study displayed that preoperative transcutaneous electric acupoint stimulation enhances quality of recovery, improves postoperative analgesia and patient's satisfaction, alleviates postoperative side effects, and accelerates discharge after general anesthesia for gynecological laparoscopic surgery.

Auricular point acupressure (APA) is a promising treatment for pain management. The pilot prospective randomized clinical trial entitled “The Anti-Inflammatory Actions of Auricular Point Acupressure for Chronic Low Back Pain” demonstrated that APA treatment affects pain perception in CLBP patients through modulation of the immune system, as reflected by APA-induced changes in serum inflammatory cytokine and neuropeptide levels.

The article of this special issue “Study on the Antinociceptive Effects of Herba Epimedium in Mice” when studied in various pain models evoked marked antinociceptive effect possibly mediated through 5-HT receptors (1A and 2A receptors) and adrenaline *β*1-receptor intervention.

The article “Efficacy of Pulsed Radiofrequency on Cervical 2-3 Posterior Medial Branches in Treating Chronic Migraine: A Randomized, Controlled, and Double-Blind Trial” showed in a randomized, double-blind, and controlled clinical trial that the pulsed radiofrequency on the cervical 2-3 posterior medial branches could provide satisfactory efficacy in the treatment of chronic migraine without obvious adverse effects.

The research article of the issue “Peripheral Neuropathic Facial/Trigeminal Pain and RANTES/CCL5 in Jawbone Cavitation” exhibited regulated on activation, normal T-cell expressed and secreted (RANTES) overexpression in silent inflamed jawbones as a possible cause for atypical facial pain/trigeminal neuralgia (AFP/TRN). Thus, the surgical clearing of fatty degenerated jawbone might diminish RANTES signaling pathways in neurons and contribute to resolving chronic neurological pain in AFP/TRN patients.

The research article entitled “The Nociceptive and Anti-Inflammatory Effects of* Artemisia dracunculus* L. Aqueous Extract on Fructose Fed Male Rats” reported that the extract of* A. dracunculus* elicited significant antinociceptive and anti-inflammatory effects in fructose fed male rats.

The research article entitled “Chinese Herbal Therapy for Chronic Tension-Type Headache” provided the evidence that Chinese herbal therapy can be clinically useful for the treatment of chronic tension-type headache.

In short, the various articles of this special issue explored the importance of each traditional/alternative way of treatment and clinical significance thus signifying that pain is best controlled using coordinated efforts using different traditional/alternate measures.

*Haroon Khan* *Haroon Khan* *Bruno Eto* *Bruno Eto* *Vincenzo De Feo* *Vincenzo De Feo* *Anwar-Ul-Hassan Gilani* *Anwar-Ul-Hassan Gilani*

